# Phase Diagram of Purified CNS Myelin Reveals Continuous Transformation between Expanded and Compacted Lamellar States

**DOI:** 10.3390/cells9030670

**Published:** 2020-03-10

**Authors:** Julio M. Pusterla, Emanuel Schneck, Rafael G. Oliveira

**Affiliations:** 1 Centro de Investigaciones en Química Biológica de Córdoba (CIQUIBIC)-Departamento de Química Biológica Dr. Ranwel Caputto, Facultad de Ciencias Químicas, Universidad Nacional de Córdoba, Haya de la Torre y Medina Allende, Ciudad Universitaria, X5000HUA Córdoba, Argentina; jpusterla@fcq.unc.edu.ar; 2 Department of Physics, Institute of Condensed Matter Physics, TU Darmstadt, Hochschulstrasse 8, 64289 Darmstadt, Germany; schneck@fkp.tu-darmstadt.de

**Keywords:** detergent-insoluble glycosphingolipid, lipid–protein domains, domain phase segregation, membrane heterogeneity, small-angle X-ray diffraction

## Abstract

Purified myelin membranes (PMMs) are the starting material for biochemical studies, from individual components up to the isolation of detergent-resistant membrane (DRM) fractions or detergent-insoluble glycosphingolipid (DIG) fractions, which are commonly believed to resemble physiological lipid rafts. The normal DIG isolation protocol involves the extraction of lipids under moderate cooling. The isolation of PMMs also involves the cooling of myelin as well as exposure to low ionic strength (IS). Here, we addressed the combined influence of cooling and IS on the structure of PMMs. The phase behaviour was investigated by small angle X-ray diffraction. Analysis of the diffraction peaks revealed the lamellar periodicity (d), the number of periodically correlated bilayers (N), and the relatives fractions of each phase. Departure from physiological conditions induced a phase separation in myelin. The effect of monovalent and divalent ions was also compared at equivalent IS, showing a differential effect, and phase diagrams for both ion types were established—Ca^2+^ induced the well-known over-compacted phase, but additionally we also found an expanded phase at low IS. Na^+^ promoted phase separation, and also induced over-compaction at sufficiently high IS. Finally, exploring the whole phase diagram, we found evidence for the direct isothermal transformation from the expanded to the compacted phase, suggesting that both phases could in fact originate from the identical primary lateral phase separation, whereas the apparent difference lies in the inter-bilayer interaction that is modulated by the ionic milieu.

## 1. Introduction

The various physical states of myelin have been described in the past, mainly based on the lamellar period (or “spacing”) d as obtained by X-ray diffraction on the peripheral nervous system (PNS). Apart from the native period, myelin can also exhibit a so-called “expanded” and a so-called “(over)compacted” phase [1,2]. In our previous studies, we described the phase separation of purified myelin membranes (PMMs) induced by cooling [3] and its relationship to detergent-insoluble glycosphingolipid (DIGs) [4,5,6,7]. In the present work, we performed a systematic study on the influence of the ionic strength (IS) on PMMs and established temperature/IS phase diagrams revealing the identity of the phases involved in the phase separation. Previously, we have shown that the phase separation of PMMs from the central nervous system (CNS) leads to a coexistence of the native period (d = 7.4–8.0 nm) with another new segregated phase. This new phase can assume two different arrangements depending on the environmental conditions: it can be (over)compacted (d < 7 nm) or expanded (d > 8.0 nm). In fact, these phases in nerve myelin have been well known since the 1970s [1,2]. Certain phase states can be promoted or suppressed by the surrounding tissue [8]. The expanded period of PNS myelin, for instance, is enhanced when connective tissues are digested in nerve. The connective tissue also turns the kinetics of phase transformations very slow. On the other hand, PMMs separated from gray matter and depleted of any surrounding connective tissue are rapidly equilibrated, mimicking the behavior of myelin in nerve and in other conditions [9].

PMMs are also the starting material for biochemical analyses ranging from individual components up to collective subfractions, such as its lipid and DIG-rich fractions. The latter have been postulated as being representative of functional lipid rafts [7]. The isolation of DIGs is performed upon cooling and it is known that temperature is a very important physical variable affecting the stability of membranes [10]. However, myelin can be also subject to other stimuli, such as bulk compression–decompression which produces diver blebs affecting myelin as a primary target [11]. Almost all classical works have aimed at differentiating between several physical states of myelin [1,2,12]. It has also been shown that myelin displaying a certain physical state can be converted into another one. This suggested the possibility of conversion from one phase to the other [1,2] although some of these conversions were shown to be irreversible. The expanded phase was also called swelled phase, mainly in the context of swelling experiments performed for phasing diffraction data and also performed for study myelin interactions [8]. These interactions were partially studied in the past as a function of ionic strength, pH [13], and the temperature in nerve [14] for native myelin. This was also before the advent of the concept of lipid rafts and DIGs. Unfortunately, in the bibliography about myelin lipid rafts [7], there is no connection to the classical X-ray diffraction studies [2]. Here, we try to fill this gap between these two areas of knowledge. Myelin is also exposed to changes in IS during purification, or in studies of inter-membrane interactions. Therefore, it is critical to know the thermal phase behavior of PMMs as well as their response to other variables. In the present work, we explored in a systematic way the combined response of CNS PMMs to temperature and ionic milieu and establish 2D phase diagrams. We selected NaCl and CaCl_2_, as they are physiologically very relevant, their interactions with myelin are mainly unspecific, and at the same time their effects are well studied for certain conditions of concentration and temperature in the classical literature [1,2] and by ourselves as researchers [3,9], showing that they can modulate the structure of myelin. Small angle X-ray diffraction was employed to identify different membrane phases from their characteristic lamellar periods [2]. This approach was non-perturbing, in contrast to detergent extraction. It moreover provided simultaneous information for the different phases regarding the lamellar spacing, degree of ordering, and relative amount of each phase.

## 2. Materials and Methods

### 2.1. Preparation of PMMs

PMMs were prepared from bovine spinal cord, which was a gift from Bustos y Beltrán S.A. abattoir (Córdoba, Argentina) under the supervision of the veterinary of sanity authority. According to Haley et al. [15], the purification protocol consists of several osmotic shocks and direct as well as inverse sucrose gradient centrifugations to discard gray matter constituents according to density. After three final rinsing steps in water, the myelin membranes were lyophilized and stored at −20 or −70 °C. Chemicals were of analytical degree, purchased from Merck (Darmstadt, Germany), and used without further purification.

PMMs retain the biomolecular composition of myelin [16] as well as the characteristic patterns of isolated myelin in TEM [3,17,18]. PMMs also exhibit an electron density profile [3] similar to that of myelin [19].

### 2.2. Small-Angle X-Ray Diffraction in Suspension

Small angle X-ray diffraction was used to determine the lamellar period (or “spacing”) d of the bilayers. Measurements were performed at the DO2A/SAXS2 beamline of the Laboratorio Nacional de Luz Sincrotron LNLS (Campinas, Brazil), with a beam energy of 8.3 keV corresponding to a wavelength *λ* = 1.488 Å. Lyophilized PMMs were suspended at 10–12 mg/mL and warmed up to 45 °C to ensure hydration. After three thawing and cooling cycles from 4 to 40 °C, the samples were stored at 4 °C until the measurements. Thus far, we have not observed any influence of the sample history on the behaviour of purified myelin.

A series of NaCl and CaCl_2_ solutions with equivalent IS was prepared. The IS was calculated from Equation (1):

(1) $$IS=\frac{1}{2}\times \sum z_{i}^{2}\times c_{i,0}$$

where $c_{i,0}$ is the concentration of ion species i at an infinite distance from the central charge, and $z_{i}$ is the valency of the respective ion, for instance z = +1 for Na^+^, z = +2 for Ca^2+^, and z = −1 for Cl^−^ [10]. The selected IS were 0, 9, 36, 75, 150, 300, 600, and 1200 mM. In addition, three NaCl solutions (375, 450, and 525 mM) in the range relevant for the continuous phase transformation were used. The phase behaviour of PMMs was investigated for various temperatures (4–48 °C) below the myelin protein denaturation temperature.

PMMs were injected into a liquid sample cell between two mica plates. The sample-to-detector distance was 1 m, calibrated with silver behenate. The exposure time for each experiment was 5 min. For radial integration of the Debye–Scherrer rings on the 2D detector (MARCCD 165), we employed the free software Fit2D V12.077 from Andy Hammersley at the European Synchrotron Radiation Facility [20].

The intensities are presented as a function of the magnitude of the scattering vector q (Equation (2)):

(2) $$q=\frac{4\pi }{\lambda }\sin\left(\frac{2\theta }{2}\right)$$

where 2θ is the scattering angle with respect to the incident beam. The corresponding lamellar periodicities d then follow from the Bragg equation as Equation (3):

(3) $$d=\frac{2\pi h}{q}$$

where h = 1, 2, is the peak order. The number N of periodically correlated bilayers under various conditions was obtained by applying the Scherrer equation [21] to the Bragg peak full width at half maximum (w) of a Lorentzian curve (Equation (4)):

(4) $$N=\frac{2\pi \times 0.88}{d\sqrt{w^{2}-r^{2}}}$$

where r is the instrumental resolution (here: *r* ≈ 0.04 nm^−1^). The diffracting power of native phase ($P_{nat}$) was defined by using the Patterson function [22] (Equation (5)):

(5) $$P_{nat}=\sum_{h}\left[\frac{hI_{nat}\left(h\right)}{d^{2}}\right]$$

where $hI_{nat}\left(h\right)/d$ is the Lorentz-corrected intensity of the reflection of order h. The integrated $I_{nat}\left(h\right)$ was estimated from the areas under the associated Bragg peaks. The fraction of myelin in the native phase is expressed as the relative diffracting power ($RDP_{nat}$), which is the ratio of the diffracting power $P_{nat}$ to the initial amount ($P_{nat}$ + $P_{non-nat}$) (Equation (6)):

(6) $$RDP_{nat}=\frac{P_{nat}}{P_{nat}+P_{non-nat}}$$

## 3. Results

**Figure 1:**
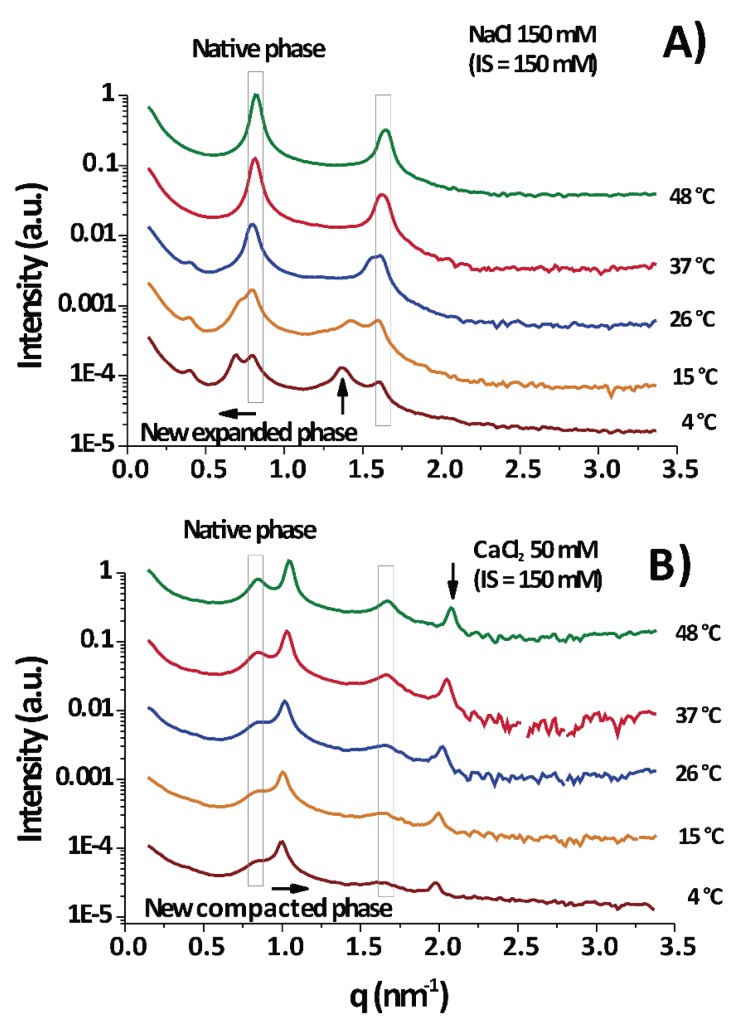
Diffraction signals of purified myelin membranes (PMMs) at different temperatures and in different aqueous conditions. (**A**) Under near physiological conditions (NaCl 150 mM) and (B) at high CaCl_2_ (50 mM, equal ionic strength (IS) to A). In (A), the major Bragg peaks of myelin (at 0.8 and 1.6 nm^−1^) were the only peaks present at 37–48 °C (surrounded in a vertical rectangle), but under cooling a new phase appeared with a peak at lower q (arrow pointing to the left), which coincided with higher d—an expanded phase. In (**B**), the native peak appeared again (inside the rectangle), and additionally a new Bragg peak at higher q appeared (≈1 nm^−1^, arrow pointing to the right)—a compacted phase. The second order peaks (h = 2) associated with the non-native phases are indicated with vertical arrows.

According to the diffraction measurements, only lamellar phases were observed, as evidenced from the integer relationship between the positions of the peak maxima (some examples are shown in Figure 1) [10,23]. The period d of the lamellar phases were calculated by Equation (3).

Under physiological conditions (NaCl 150 mM/150 mM IS) PMMs normally show a series of major diffraction peaks (enclosed in vertical boxes in Figure 1) located at ≈0.8 and ≈1.6 nm^−1^ [24]; they correspond to a membrane period of d = 7.7 ± 0.2 nm. Consequently, these are the only peaks in Figure 1A at 37–48 °C. It is seen that the native period (vertical box) was present in almost all temperature–IS conditions.

Upon cooling to 15–4 °C (DIGs isolation temperature), the native peak split into two peaks, the new one shifted to lower q as the temperature decreased. This shift to lower q reflected the expansion of the lamellar period of the membranes.

Figure 1B (CaCl_2_ 50 mM/150 mM IS) shows a permanent split of the peak into the native period and a non-native period, but the peak position of the latter shifted in the opposite direction (as compared to Figure 1A), to a higher q and consequently revealed a compaction of the membrane stack. Figure 1A,B are at equal IS and a differential effect of ions is evident. As a conclusion, Figure 1 shows two examples of measurements taken each at different temperatures for two particular cases: one for myelin experiencing expansion under cooling in physiological conditions (Figure 1A) and other one experiencing (over)compaction (Figure 1B) with high CaCl_2_ concentration. The new major peaks can appear at low q (q < 0.8 nm^−1^), in which case the phase is called separated, expanded, or partially swelled, with periods ranging from 8.5 to 11 nm or more. Alternatively, the new peak can appear at q > 0.8 nm^−1^, more precisely around 1.0 nm^−1^, in which case the phase is called (over)compacted and this is clearly a lipid-enriched phase [25], with only d = 6.6 ± 0.4 nm, depleted of proteins [2]. These different non-native phases are well known and observations of them are found overall in the myelin literature [2,3].

### 3.1. Spacings of the Different PMM Phases

**Figure 2:**
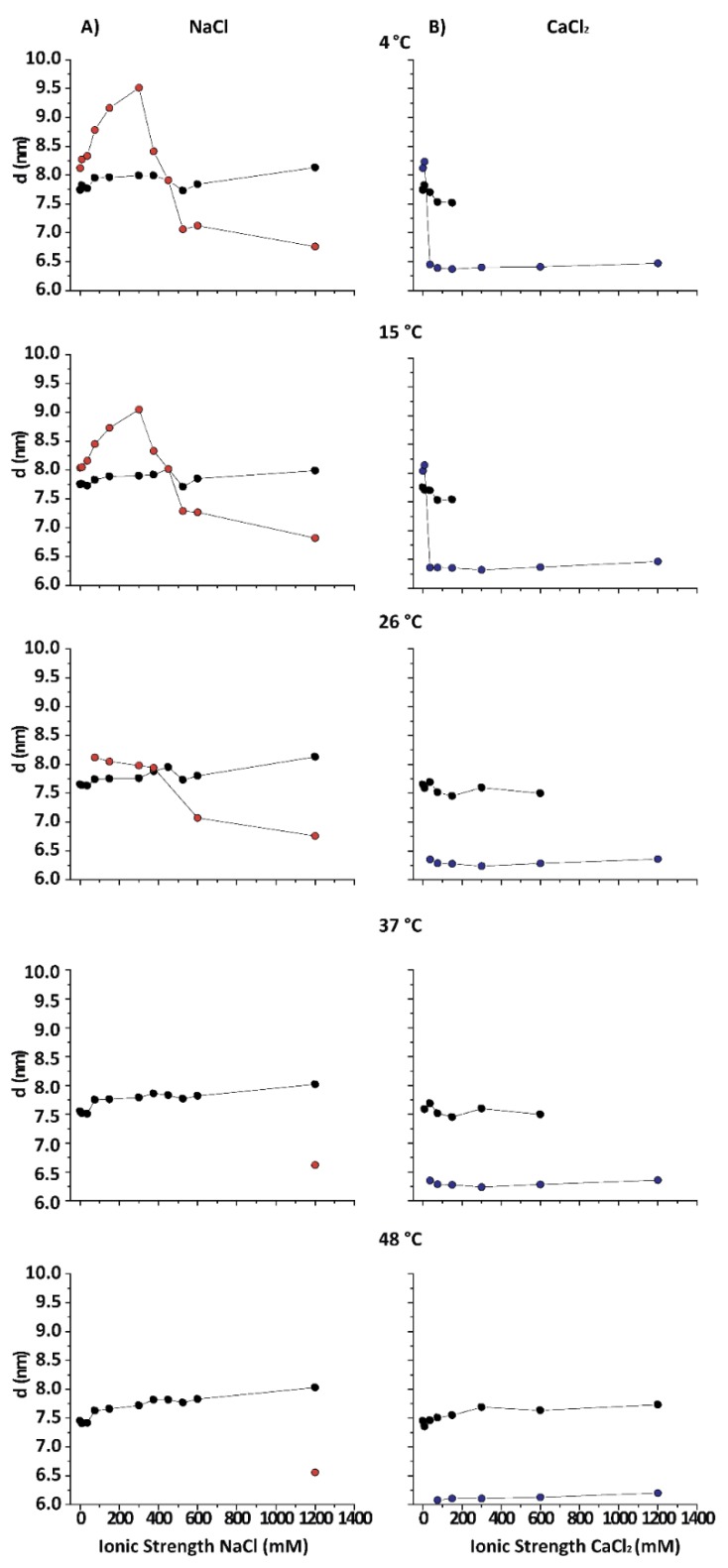
Periodicities (d) for PMMs phases under various temperature and IS conditions: (**A**) for NaCl solutions and (**B**) for CaCl_2_ solutions; the numbers in panels refer to temperature. The nearly horizontal lines (black symbols) at *d* ≈ 7.4–8.0 nm indicate the native phase. Upper values are traditionally defined as the expanded, separated, or partially swelled phase, and lower values correspond to the (over)compacted phase. Several measurements, but not all, were taken in two independent repetitions, and the error was estimated to be between ≈0.2 nm (compacted and native phases) and ≈0.3 nm (expanded phase). Black dots: native phase; red dots: non-native phase in NaCl; blue dots: non-native phase in CaCl_2_.

Figure 2 shows the lamellar periods in PMMs as a function of the IS for various temperatures in NaCl- and CaCl_2_-based solutions. The native period (black symbols) is present at all temperatures in NaCl-based solution. At 26 °C, the obtained results are consistent with what is reported in the literature for room temperature conditions [13]. At 15 and 4 °C (DIG isolation temperature) the behaviour of purified CNS myelin is nevertheless very different. Upon addition of NaCl (from 0 to 0.3 M), first an increasingly expanded phase was observed (red symbols), which contracted in the 0.375–0.45 mM range and then (at NaCl > 0.5 M) collapsed to d value characteristic for the (over)compacted phase, which was only marginally sensitive to a further increase in the NaCl concentration. Relatively low IS and temperature led to an expanded phase. Up to 3 mM CaCl_2_ (9 mM IS) led to a slightly expanded phase (d ≈ 8.3 nm) at low T (blue symbols), undistinguishable from the phase expanded by NaCl at the same IS. Nevertheless, as the IS increased, the behaviour of myelin in NaCl and CaCl_2_ solutions rapidly diverged. Previous works [12,25] had already shown that CaCl_2_ induces a cooperative compaction above 12 mM (36 mM IS, see Equation (1)) in nerve myelin, and a similar effect is seen here on PMMs. At extreme conditions, (high CaCl_2_ and low temperature) the native phase completely disappeared. On the other hand, in the presence of NaCl at 4 °C, the expanded phase persisted up to much higher salt concentrations (up to 450 mM IS), which indicated a specific role of Ca^2+^ in the stabilization of the (over)compacted phase [25]. In other words, both salt types were able to promote the expanded phase as well as the (over)compacted phase, but at very different concentration thresholds. It should be noted that this difference between Na^+^ and Ca^2+^ persisted also when the IS was considered instead of the concentration. In other words, the effect must be considered ion-specific or at least dependent of the cation valence. As a general conclusion, the states of myelin under extreme conditions (i.e., at the lowest and highest salt concentrations) were qualitatively the same for CaCl_2_ and NaCl. However, at intermediate (near-physiological) salt concentrations, the CaCl_2_ shifted the balance more towards (over)compaction, whereas NaCl shifted it more towards expansion.

### 3.2. PMMs Phase Diagrams as a Function of Temperature and IS

**Figure 3:**
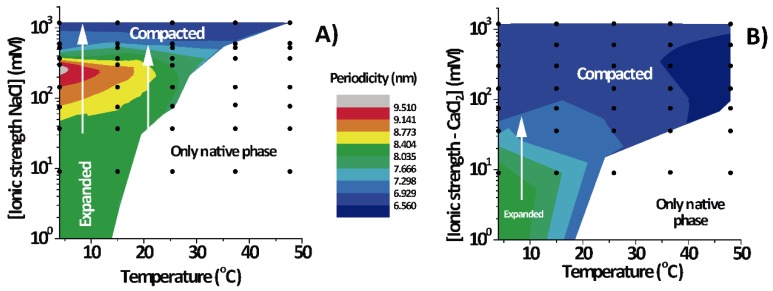
Lamellar period of the non-native phases of PMMs as a function of temperature and IS in aqueous salt solutions based on NaCl (**A**) and CaCl_2_ (**B**). The white regions designate sections of the phase diagram in which only the native phase existed. The vertical arrows represent isothermal paths for the transformation expanded into (over)compacted states, and the black dots are the measured data points for each temperature and IS.

Figure 3 shows the lamellar period of the PMMs’ non-native phase as a function of temperature and IS for solutions based on NaCl (Figure 3A) and on CaCl_2_ (Figure 3B). The white region designates conditions under which only the native phase is present. As can be seen, the expanded and (over)compacted phases were both present in CaCl_2_ as well NaCl solutions but at different IS values. The most striking feature of the phase diagram is the continuous transformation of the non-native period from expanded to compacted, as indicated with vertical arrows in both panels. The transition can be induced isothermally, by variation of the NaCl. On the other hand, such a direct transformation from the expanded into the (over)compacted state without passing through a native phase intermediary cannot be observed in thermal scans at constant salt concentration (which would correspond to a horizontal path in the phase diagrams). This is likely the reason why the direct connection between the two non-native phases had not been identified in the classical literature [2].

### 3.3. Quantification of the Relative Fraction of Each Phase

**Figure 4:**
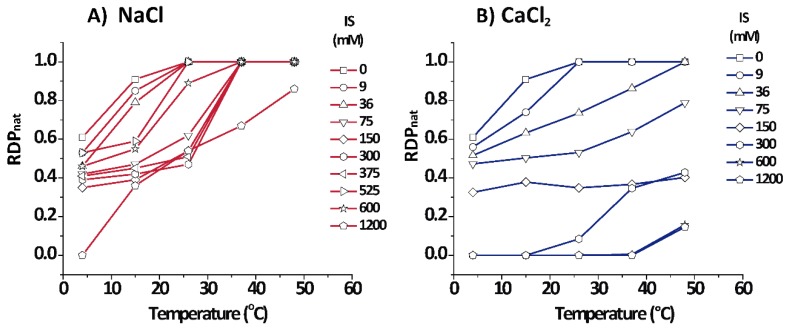
Relative diffracting power ($RDP_{nat}$) of the native phase as a function of temperature and IS. Data for PMMs in aqueous salt solutions based on NaCl and CaCl_2_ are shown in panels (**A**) and (**B**), respectively.

Figure 4 shows the relative amount for each phase as estimated from the relative diffracting power ($RDP_{nat}$) of each phase (see Equations (5) and (6)) for PMMs in aqueous salt solutions based on NaCl and CaCl_2_. Cooling shifted the equilibrium to the non-native phase for both salt types, as seen from the lower $RDP_{nat}$ of the native phase. On the other hand, in NaCl solutions, heating promoted the increment of the native phase up to a point at which $RDP_{nat}$ = 1.0, except for an extreme condition of 1.2 M IS where some (over)compacted phase persisted. In contrast to NaCl, CaCl_2_ quickly shifted the equilibrium in the direction of non-native phases, mainly towards the (over)compacted phase.

### 3.4. Number (N) of Periodically Correlated Bilayers

An interesting point apart from the relative amount of each phase is how well they are periodically arranged into stacks of membranes. The number of periodically correlated bilayers (N) can be obtained from the width of the diffraction peaks (see Equation (4)).

**Figure 5:**
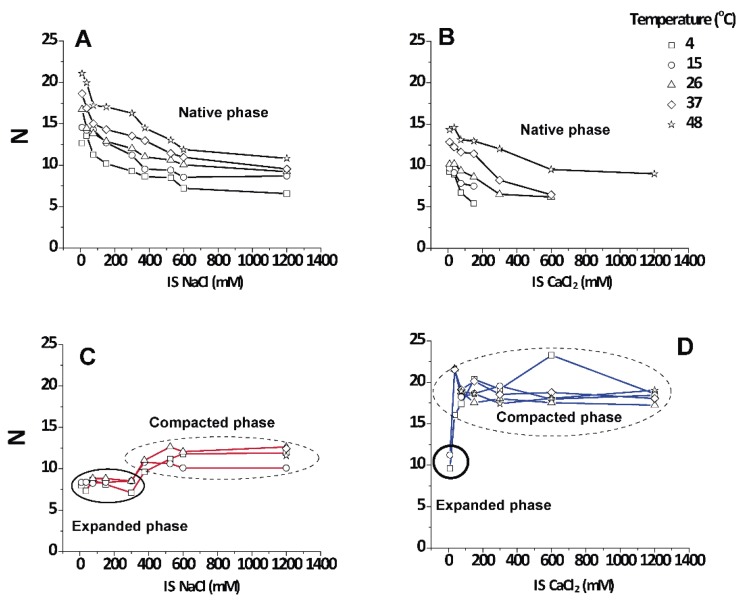
Number of periodically correlated bilayers (N) as a function of IS and temperature for the different phases present in PMMs. The graphics show N for the native phase in NaCl (**A**) and CaCl_2_ (**B**), and for the non-native phases in NaCl (**C**) and CaCl_2_ (**D**). Squares: 4 °C, circles: 15 °C, triangles: 26 °C, diamonds: 37 °C; stars: 48 °C.

Figure 5 shows that the native period myelin was highly variable and grew up to a limiting value of 12–20 lamellae in the direction of heating as the non-native phases disappeared. Irrespective of the salt type, *N* decreased with increasing IS. It is known that CNS myelin persists in a packed state, even in pure water (IS = 0), which is in contrast to PNS myelin that swells and loses its lamellar correlations [13]. For the non-native periods, the data grouped into two distinct regimes that were not very responsive to temperature (encircled areas in Figure 5C,D). The values for the expanded period are about N ≈ 9 ± 2 correlated bilayers, which agrees with the well-known transverse stacking of membrane domains in myelin membranes [13]. The (over)compacted phase is more highly correlated, with *N* = 10–20, as was reported earlier [25]. These observations led us to conclude that the non-native phases, no matter whether they are induced by NaCl or CaCl_2_, are largely non-responsive to environmental conditions once they are nucleated (encircled in Figure 5C,D), although the (over)compacted phase induced by CaCl_2_ appeared to be somewhat more strongly correlated.

### 3.5. Non-Monotonic Dependence of Non-Native Phase Lamellar Period

**Figure 6:**
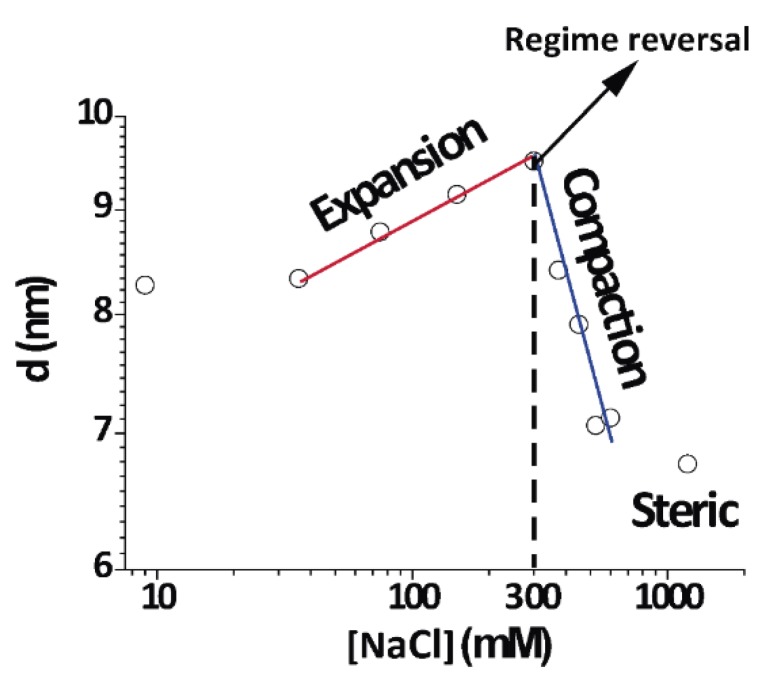
Periodicity (d) as a function of the IS in double logarithmic plot at 4 °C. The IS dependence of *d* was clearly non-monotonic and exhibited distinct regimes, indicated with straight lines.

An important finding in this work was the direct and continuous transition between the expanded and compacted phases of PMMs, which was salt-induced and best observed at 4 °C. Figure 6 shows an isothermal cut at 4 °C for different NaCl concentrations (same data as Figure 2A at 4 °C) in a double-logarithmic representation. As can be seen from the distinct linear regimes in this representation, different power laws seemed to characterize the initial expansion (at low NaCl concentrations), the compaction (at intermediate NaCl concentrations), and finally a plateau at high NaCl concentrations, when no further compaction was possible due to short-range repulsive interactions. In contrast to this complex behaviour, the native period of myelin was rather insensitive to the salt concentration because its period was dictated by the protein components [2].

This is the very first report about this behaviour in myelin. Previous studies have revealed a swollen or expanded period as well a native period at room temperature and 0.1–0.2 NaCl IS, consistent with what was found at 26 °C [13]. We expanded such a work in a systematic way and showed that the expanded myelin period contracted under increased IS. At 15 and 4 °C, the expanded phase further expanded up to concentrations of 0.3 M NaCl. This region of the phase diagram very clearly showed continuity or direct transformation between the expanded and compacted phase.

## 4. Discussion

A major result of this work was the observation of a direct continuous transition between the two non-native phases of PMMs without passing through the native phase. The transition was continuous in the sense that intermediate values between the (over)compacted and the expanded spacings could be found (Figure 2). The same applied to the evolution of the RDP shown in Figure 4. Regarding these two observables, the transition clearly was not an “all or nothing” process. However, regarding the number of correlated membranes shown in Figure 5, the picture was somewhat different, because the transition there rather appeared as a step-like change, as was previously shown for the transition from native to (over)compacted myelin in the old literature [25]. This transition did not occur at physiological or higher temperature, but was relevant for DIG extraction at the low temperature. Importantly, the continuous transition was controlled by the IS along an isothermal and could not be induced by temperature variation and constant IS. It is also interesting to note that close to 300 mM [NaCl] (maximum in Figure 6), there was a change in behaviour from expanding to compacting regime as a function of [NaCl]. This was evident at 4–15 °C, but an increase of temperature towards the physiological range weakened this effect (Figure 2 at 26 °C). Further increase in the temperature eventually transformed the non-native phases into the native phase.

Another remarkable fact is that during the transformation from expanded to compacted spacing at 4–15 °C, the $RDP_{nat}$ of the native phase (Figure 4) did not cross through a minimum but it displayed a monotonic step-like behaviour. This meant that the expanded phase did not transform first into the native (otherwise $RDP_{nat}$ should have been 1) and then into the (over)compacted phase, but rather it transformed directly from the expanded into the (over)compacted phase. A similar monotonic behaviour in line with this picture could also be observed with the number of correlated bilayers of the native state.

The presence of salt typically leads to a screening of electrostatic repulsion when the membrane surfaces carry a non-negligible surface charge density. In this case, one expects a monotonic decrease of the lamellar period with increasing salt concentration. However, favourable interactions of certain ions with the surfaces can result in an effectively enhanced charge density and, in turn, stronger repulsion. The strength of this effect in general depends on the ion type and on the surface chemistry and can strongly deviate from the usual Hofmeister series [26]. For example, an increase in the lamellar period of neutral phosphatidylcholine (PC) lipid membranes by the addition of salt is well documented and has been studied best for calcium ions, which exhibit preferential interactions with PC [27,28,29,30,31]. Increasing the CaCl_2_ concentration not only increases the surface charge density but also the screening effect [31], so that also the repulsion is reduced. In this case, a non-monotonic behaviour, as in our data in Figure 6, is expected because the surface charge density will saturate at some point, whereas the screening strength further increases as the square root of the concentration. The concentration at the maximum repulsion as well as the precise repulsion strength depend on the magnitude of the ion affinity and details of the interfacial force balance. In this light, and considering additional factors such as a potential influence of the ions on the area per lipid and on the bending rigidity, we refrained from modelling the experimental data on a quantitative level. Regarding the affinity of ions for the membrane surfaces, we note that neither Na^+^ nor Cl^−^ had significant affinities for commonly studied PC lipids. Myelin membranes, however, display various chemical motifs including glycolipids, which can exhibit preferential interactions with monovalent and divalent ions, as was recently shown [32].

As just discussed, the crossover from expansion to compaction upon IS variation (at ≈300 mM) at low temperature points leads to a near-critical state of myelin under physiological conditions regarding inter-membrane interactions. The change in the interlamellar water amount upon variation of the lamellar period by Δd can be roughly estimated when assuming a constant membrane thickness. In this case, Δd directly translated into a change in the equivalent number $\Delta n_{l}=\;\Delta d/l_{w}$ of “water layers”, where $l_{w}={\left(v_{w}\right)}^{1/3}$ =0.31 nm is the linear dimension of a water molecule of partial molecular volume, $v_{w}=0.030\;{\mathrm{nm}}^{3}$. This procedure yielded $\Delta n_{l}=10$ between the lamellae in the more extreme calculation; this was considering full expansion in NaCl at 4 °C and taking the most compacted state (CaCl_2_, at the same temperature). More conservative calculations (taking NaCl also for the most (over)compacted state) rendered $\Delta n_{l}=8$ for the highest hydration.

In the present work, we showed that in CNS these non-native phases related to DIGs [3] can in fact be interconverted directly without passing through the native state. This result suggests that the expanded and compacted phases are based on the same lateral phase separation that can be observed also in Langmuir monolayers [33]. This phase separation is mainly due to the insolubility of many transmembrane proteins on cholesterol enriched phases, which is further increased upon cooling, and is related to the cholesterol-enriched phase present in PMM Langmuir monolayers [33]. In multilayers, the inter-membrane interactions then additionally induce changes in the lamellar period according to the temperature and IS conditions (see vertical arrows in Figure 3). It is well known that the compacted phase is a lipid-enriched, protein-free lateral portion of the membrane; this phase can be so compact only because of the absence of proteins that dictate the period of native myelin [2]. The same lipid-enriched phase has already been observed in Langmuir monolayers [34,35]. It might thus be that the expanded phase represents the lipid-enriched phase, but in an environment that favours repulsion. In fact, it has also been shown that protein-depleted phases of myelin can exhibit more expanded periods under certain conditions [12]. In the literature, it was suggested that both expanded and compacted phases share the same electron density profile, at least at low resolution [36]. Furthermore, our group has previously shown that the DIG fraction from CNS PMMs can be compacted (at 20 mM CaCl_2_) or expanded (at 150 mM NaCl), mimicking the non-native phase of whole myelin under the corresponding environmental conditions [3].

This combined evidence lets us postulate that a single DIGs phase is on the basis of both the expanded phase and the compacted phase.

## 5. Conclusions

Our main result is that the compacted and expanded phases are closely related to each other. In fact, a continuous transformation from one phase to the other can be clearly observed at low temperature in the phase diagram. That is, this transformation took place without any detectable discontinuity in spacing and directly from the expanded to the compacted phase without passing through the native phase. This isothermal transformation followed a power law in the inter-membrane distance as a function of the IS. Thus, one diffraction peak was continuously shifted into the peak position classically assigned to the other phase. The sensitivity of the inter-membrane interaction to the ionic environment produced differential stacking of the non-native phase(s), and this fact gave origin to the differential nomenclature commonly used for the non-native phase(s) of myelin adopted when this interconnection was unknown. This shift can only be observed below room temperature in combined phase diagrams such as the ones presented here.
